# The hepatoprotective effect of livergol microemulsion preparation (nanoparticle) against bromobenzene induced toxicity in mice

**DOI:** 10.1016/j.toxrep.2019.05.005

**Published:** 2019-05-09

**Authors:** Azin Kalantari, Anayatollah Salimi, Heibatullah Kalantari, Jalal Ebrahimi Broojeni, Iran Rashidi, Atefeh Raesi Vanani, Ildikó Bácskay

**Affiliations:** a Nanotechnology Research Center, Department of Pharmacology and Toxicology, Ahvaz Jundishapur University of Medical Sciences, Ahvaz, Iran; b Faculty of Pharmacy, Department of Pharmaceutical Technology, University of Debrecen Health Science Center, Debrecen, Hungary; c Nanotechnology Research Center, Department of Pharmaceutics, Ahvaz Jundishapur University of Medical Sciences, Ahvaz, Iran

**Keywords:** Microemulsion of milk thistle, Iivergol, Liver, Bromobenzene, Mice

## Abstract

Livergol (LG), which is the extract of *Silybum marianum* and commonly known as milk thistle possess hepatoprotective effect and have got licensed for sale in Iran and other countries. LG was evaluated for its capacity to counteract the toxic effects of bromobenzene (BB) on mouse liver. The bioactive component of this plant is known to reinforce naturally occurring liver function through antioxidant activity, the stimulation of bile production and regeneration by the liver organ, resulting in enhanced protection against toxicants, hepatitis, and cirrhosis. The major bioactive components of this product are the flavonolignan ssilibinin, silidianin, silicristin, and isosilibinin. Mice were treated for 10 days with daily gavage of microemulsions (MEs), into which 0–400 mg/kg LG was dispersed. 0.36 ml/kg BB was injected intraperitoneally (ip) to each animal on day 10, followed by sacrifice on day 11, and histological evaluation of hematoxylin-eosin (HE)‐stained liver tissue samples, afterwards followed by evaluation liver enzymes level, aminotransferase (AST), alanine aminotransaminase (ALT) and alkaline phosphatase (ALP) activities. Significant suppression of BB-mediated damage to liver tissue, and increased in AST, ALT, and ALP level was observed to occur dose-responsively with LG administration, suggesting a use for LG as a chemoprotectant for persons chronically exposed to industrial solvents.

## Highlights

• Livergol (LG), which is the extract of *Silybum marianum* and commonly known as milk thistle possess hepatoprotective effect.
• Orally administered LG significantly suppresses Bromobenzene (BB)-induced increases in serum activity of enzymes AST, ALT, ALP.
• Treatment with LG has improved hepatic damages due to BB severe degeneration and vacuolation of hepatocytes.
• Based on the results the efficacy of LG in MEs showed better drug solubility and permeability which lead to improve drug absorption among different biological membranes.
• The hepatoprotective effect of this formulation against BB toxicity has been conducted through the control release, high diffusion and absorption rates and improve and increase in oral bioavailability of active pharmaceutical agents.

## 1 Introduction

Dramatic increases in use of herbal formulations for the prevention and long term management of a wide range of diseases have occurred during the past several years. This is partly due to a combination of market-driven factors, including lower cost and, in many cases a lower incidence of adverse effects in comparison to pharmaceutical drugs. Added to this, government regulatory constraints on the manufacturing and sale of naturally occurring agents is often less stringent than with other medical products.

Healthcare providers often find that patient compliance in adherence to a particular course of treatment may be substantially better when natural products, particularly extracts of medicinal plants extracts, are part of the regimen. Nevertheless, the increasingly widespread integration of such products into “mainstream” medicine provides an incentive to conduct stringent toxicological analyses on these agents incidental to their clinical use.

The present investigation evaluates the capacity of LG, a commercially available product made from extract of *Silybum marianum* (milk thistle) fruit, to protect mouse liver from acute damage by BB, a highly toxic organic solvent. previously, LG has shown efficacy in protection of the liver against toxicant exposure [1], acute and chronic hepatitis [2], and ncirrhosis [3]. It has also been shown to directly promote hepatic tissue regeneration, increasing production of bile and endogenous antioxidants [4].

In these experiments, mice were treated with selected dosages of LG and were challenged with BB, also known as bromobenzol, a highly toxic organic solvent. The primary mechanism of its hepatotoxicity occurs as a result of hepatic phase I metabolites produced during initial degradation of the compound by liver cells [5,6]. Further degradation of these compounds produce hepatic phase II products, such as bromophenolisomers, which are highly nephrotoxic [7], and may cause kidney damage.

Authors of this report have previously demonstrated the nephroprotective capacity of *Cassia fistula* (golden shower tree) fruit in BB-exposed mice [8], the mutagenic effects of *Artemisia dracunculus* (Tarragon), a widely used dietary herb [9,10], and Dillsun, an oleo extract of the herb *Anethumgraveolens*, commonly used in Iran to improve liver function [11]. Previous research by these authors has further demonstrated the dermal protective effects and toxigenic profiles of quince seed mucilage [12], and of sour cherry seed oils and flavonoids [13,14].

The aim of this work is to find out the toxicological profile of milk thistle that may aid in its clinical use by healthcare professionals. Mice were administered selected dosages of a microemulsion of milk thistle extract (LG). Subsequently, they were challenged with BB, and evaluated for pathological elevation in serum levels of three liver enzymes, known to increase in response to hepatotoxic influences, and for adverse effects on liver tissue integrity. The extent to which both of these outcomes were suppressed in LG-treated animals is an indicator of the protective and therapeutic efficacy of the plant in response to hepatotoxins.

## 2 Materials and methods

### 2.1 Animals

Swiss albino male mice were used in these experiments with an average weight of 25 ± 5 g. They were fed regular rodent chow pellets *ad libitum* with free access to tap water and were maintained at an ambient temperature of 25 ± 2 °C, with a relative humidity of 55 ± 5%, and a 12 -h light-dark cycle. These animals were acclimatized for one week prior to initiation of experiments. The mice were handled and received humane care in compliance with the principles of laboratory animal care formulated by the National Society for Medical Research and Guide for the Care and Use of Laboratory Animals prepared by the United States National Academy of Sciences, and published by the U.S. National Institutes of Health (Publication No. NIH 85-23, revised in 1996). All the protocols for their use in this investigation were approved by the institutional review board (IRB) of Ahvaz Jundishapur University of Medical Sciences (approval number: u-91134).

### 2.2 Microemulsions preparation

**Fig. 1:**
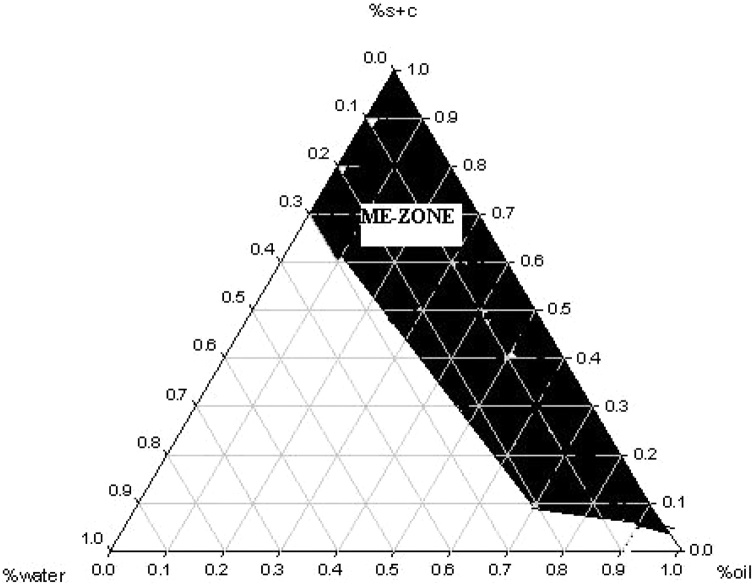
Pseudo-ternary phase (PTP) diagram of the system (olive oil; Tween 80: Span 20; propylene glycol/ water). Transparent MEs are represented by the dark area, with the remaining sectors of the PTP diagram representing cloudy (turbid) emulsions.

The microemulsions vehicle containing LG was dispersed for oral gavage administration to the mice was prepared from a system composed of olive oil (commercial grade, purchased locally in Iran), Tween 80 (Sigma-Aldrich, St. Louis, MO, USA), and Span 20 (Sigma-Aldrich) (1:1 ratio), as surfactant and co-surfactant and propylene glycol (Sigma) (3:1 ratio of surfactant/co-surfactant). Pseudo-ternary phase diagram, presented in Fig. 1, were plotted to discover the presence of different ME regions.

Livergol (0.5%.1%,2%and 4%) was added to oil phase and then S/Co mixture and a suitable amount of double distilled water were added to the mixture drop wise and continued by stirring the mixtures at ambient temperature until a uniform mixture was obtained. The components of the suitable formulation are 31% oil, 34% surfactant-cosurfactant, and 35% water.

### 2.3 Droplet size examination

The mean droplet size of MEs were obtained using by SCATTER SCOPE 1 QUIDIX (South Korea) at 25 °C.

### 2.4 pH and viscosity measurement

The viscosities and the pH values of the MEs were measured using by Brookfield viscometer (DV-II + Pro Brookfield, The USA, with a shear rate of 100 rpm and pH meter (Mettler Toledo SevenEasy, Switzerland) at 25 °C, respectively.

### 2.5 Drugs, stimulants, and control reagent (vehicle)

Livergol, which is an extract of *Silybum marianum* (milk thistle) was purchased from an Iranian manufacturer (Barich Herbal Pharmaceuticals, Kashan, Iran). The component profile for this sample may be obtained on request from the manufacturer (Barich). The preparation of the extract for use as in the present study was conducted at the Department of Pharmacognosy of the School of Pharmacy, Ahwaz Jundishapur Medical Science University. Briefly, 300 g of powdered milk thistle extract was extracted in 90% ethanol for 3 days, and filtered. The filtrate was re-extracted with 90% ethanol three times. The pooled filtrate was concentrated to dryness using a vacuum evaporator (Adolph, Model 462, Germany). The dried extract was suspended in normal saline (NS) and prepared for administration to mice, as previously described [15].

The composition of the LG formulation used in the present study was adjusted to approximately the same range of component composition as commercially available products marketed as a nutraceutical. A typical human single dose (in tablet form) contains 70 and 140 mg of silymarin per tablet [16]. The main ingredients of this product are flavonolignan of *S. marianum*, such as silibinin, silychristin, silydianin, and 2–3 dehydrosilybin derivatives, collectively in whole fruit extract form called silymarin. The MEs vehicle without LG was administered to mice as a negative control treatment. BB was purchased from Sigma (St. Louis, Missouri, USA). Chemicals (methanol, ethanol, formalin, paraffin, and salts) were purchased from the local market in Iran. Before administration of BB to mice, the compound was dissolved in liquid paraffin (1/2 v/v, respectively).

### 2.6 Treatment group design, drug administration, and sample collection

Mice were randomly assigned to eight test groups of ten animals each, based on LG dosage. To determine the existence of a dose-responsive relationship between the quantity of LG administered and hepatotoxicity, members of each group were administered LG-supplemented MEs treatments daily by oral gavage for 10 days, in a dose range of 0–400 mg/kg LG body weight of each animal. On the 10thday of the study, 1 h following the last dose of LG, each animal received ip injections of BB at a dosage of 0.36 ml/kg, body weight.

The groups were segregated according to the following treatment combinations: GROUP 1 negative control animals (saline gavage): LG =0 mg/kg, BB = 0; GROUP 2 (ME vehicle gavage): LG =0 mg/kg, BB = 0; GROUP 3 (MEs gavage): LG =400 mg/kg, BB = 0;GROUP 4 (MEs gavage): LG =0 mg/kg, BB =0.36 ml/kg, ip; GROUP 5 (MEs gavage): LG =50 mg/kg, BB =0.36 ml/kg, ip; GROUP 6 (MEs gavage): LG =100 mg/kg, BB =0.36 ml/kg, ip; GROUP 7 (MEs gavage): LG =200 mg/kg, BB =0.36 ml/kg, ip; GROUP 8 (MEs gavage): LG =400 mg/kg, BB =0.36 ml/kg, ip.

24 h after the last dose (on the 11thday), mice were anesthetized with sodium pentobarbital (80 mg/kg, i.p) and sacrificed under anesthesia, followed by sample collection. Blood was collected from the jugular vein and placed at ambient room temperature (approximately 25^0^C) for 40 min to allow clot formation. Defibrinated serum was extracted by centrifugation for 10 min at 2500 rpm. The livers were excised immediately after collection of blood, weighed, washed in ice cold saline, and stored in 10% neutral phosphate-buffered formalin for histological examination.

### 2.7 Analysis of samples for hepatotoxicity

5-μm thick histological sections were prepared from each liver sample, HE‐stained, and observed under a light microscope to evaluate the extent of BB-associated microstructural tissue damage in each dosage group, as described in previous studies by these authors [9,13,17]The toxicogenic effects on liver metabolism were assessed by measurement of the activities of 3 hepatic enzymes known to increase in response to toxic insult: AST, ALT, ALP. They were assayed in mouse serum by the method of Reitman and Frankel [18], and ALP activity was estimated using King’s methodology [19].

### 2.8 Statistical analysis

All data are presented as mean ± standard error of mean (SEM). Statistical analysis was carried out with SPSS software, performing one-way ANOVA, followed by Tukey’s post-hoc test. Probability values <0.05 were considered the threshold for significant difference between the magnitude of differences between test groups versus negative control animals with respect to enzyme activities. Significance (*p*) values are shown for comparison of average enzyme activities for each LG-treated group with animals treated with BB only. P < 0.05 is the threshold for significant difference between groups with respect to each outcome variable.

## 3 Results

### 3.1 Characterization of the microemulsions

The mean droplet sizes of ME formulations in ratios of 0.5%, 1%, 2% and 4% are 26.3 ± 0.9 nm, 19.5 ± 0.3, 25.3 ± 0.7 and 20.8 ± 0.7 nm, respectively. The ME formulations had the average viscosity of 50 ± 1 centipoises, and the average pH of 5.6 ± 0.2.

### 3.2 Serum liver enzyme activities

The outcome of assays for serum liver enzyme activities in each dosage group of animals is shown in Table 1. As expected, the highest levels of AST, ALT, and ALP activation was observed in the blood of animals in Group IV, which had been administered 0.36 mg/kg of BB but no LG. The activities of each of these three enzymes in Group IV mice, which constitute positive controls, were significantly higher than corresponding activities in any of the other groups (p < 0.05). Additionally, a trend for dose-responsive decrease in each enzyme was observed in animals receiving emulsions supplemented with LG in the dose range of 50 mg/kg (Group V) through 400 mg/kg (Group VIII) (p < 0.05). Finally, it was observed that no significant differences in the activities of any of the enzymes were observed in the comparison of Group I animals, which were administered NS gavage with corresponding activities in Group II mice, which were treated with the “blank” MEs vehicle.

**Table 1:** Serum hepatic enzyme activity levels. Serum activities of AST, ALT and ALP in mice treated for 10 days with gavage of NS, MEs vehicle (base), or MEs with 400 mg/kg LG, and ip injections of 0.36 ml/kg BB, plus 50, 100, 200, and 400 mg/kg LG (dispersed in MEs).Enzyme activity levels are expressed in International Units (IU) per liter of serum ± SEM. Significance (p) values are shown for comparison of average enzyme activities for each LG-treated group, including animals treated with BB only (Group IV).*P* <  0.05 is the threshold for significant difference between groups with respect to each outcome variable.

Treatment Group(n = 8 per group)	AST(IU/l ± SEM)	ALT(IU/l ± SEM)	ALP(IU/l ± SEM)
**Group I**	226.70 ± 8.22^b^	68.90 ± 7.18^b^	175.40 ± 7.36^b^
NS vehicle, 0 mg/kg LG, 0 mg/kg BB			
**Group II**	231.90 ± 5.64^b^	72.10 ± 6.40^b^	172.90 ± 7.14^b^
MEs vehicle, 0 mg/kg LG, 0 mg/kg BB			
**Group III**	223.5 ± 10.61^b^	67.20 ± 6.89^b^	171.50 ± 8.37^b^
MEs vehicle., 400 mg/kg LG, 0 mg/kg BB	P = 0.032	P=0.027	P = 0.031
**Group IV**	371.50 ± 27.15^a^	128.70 ± 11.50^a^	252.60 ± 12.16^a^
MEs vehicle, 0 mg/kg LG, 0.36 mg/kg BB			
**Group V**	347.10 ± 27.50^a^	119.90 ± 8.25^a^	235.30 ± 7.46^a^
MEs vehicle, 50 mg/kg LG, 0.36 mg/kg BB	P=0.06	P=0.066	P = 0.059
**Group VI**	339.60 ± 25.43^a,b^	110.50 ± 9.37^a,b^	223.6 ± 7.7^a,b^
MEs vehicle, 100 mg/kg LG, 0.36 mg/kg BB	P=0.030	P=0.034	P = 0.039
**Group VII**	290.50 ± 16.24^a,b^	92.70 ± 10.91^a,b^	197.00 ± 11.03^a,b^
MEs vehicle, 200 mg/kg LG, 0.36 mg/kg BB	P = 0.032	P=0.029	P = 0.032
**Group VIII**	244.90 ± 12.59^b^	85.10 ± 8.49^a,b^	190.70 ± 9.58^a,b^
MEs vehicle, 400 mg/kg LG, 0.36 mg/kg BB	P=0.036	P=0.038	P = 0.028

*p < 0.05 in comparison with drug-free, saline-treated, negative control animals (Group I). **Significance of comparison with mice treated with BB, but not LG (Group IV). (a) Significance difference with control group (*P* <  0.05). (b) Significance difference with positive control (*P* <  0.05). (a, b) significance difference with control group and positive control (*P* <  0.05).

### 3.3 Effects of BB and LG on liver histopathology

**Fig. 2:**
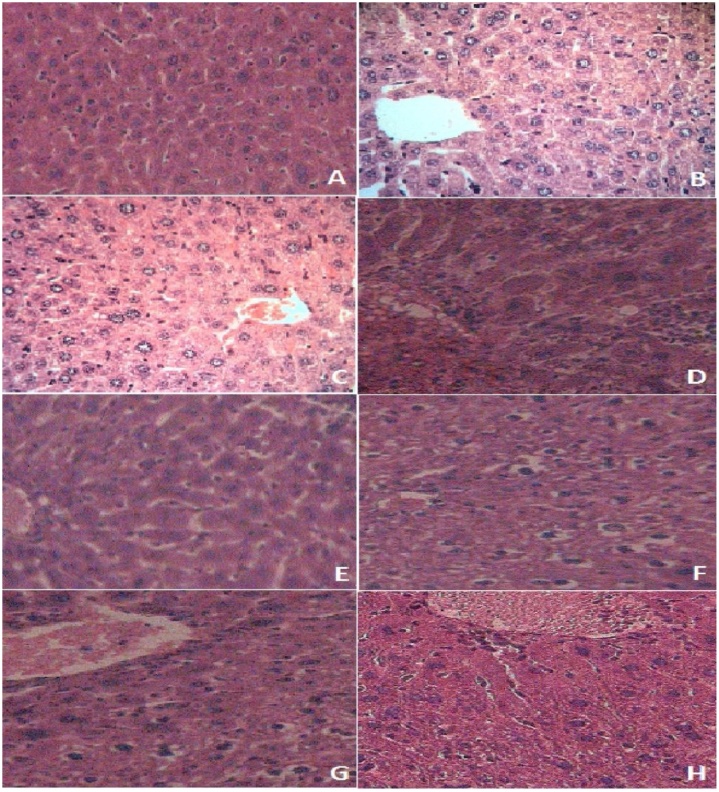
Histopathological evaluation of liver tissue.5-μm thick tissue sections harvested from Swiss Albino mice, HE-stained and formalin-fixed, and examined by light microscopy. Results shown correspond to hepatic tissue taken from animals treated by oral gavage for 10 days with NS or MEs vehicle, with or without selected dosage of LG, followed by ip injection of 0.36 ml/kg BB or vehicle (MEs without LG), and sacrifice 24 h after injections. Photographs at 400X magnification are shown for tissue from animals receiving the following treatment combinations: (**A**) NS, no BB; (**B**) MEs vehicle, no BB; (**C**) MEs with 400 mg/kg LG, no BB; (**D**) MEs vehicle, no LG, 0 0.36 ml/kg BB; (**E**) MEs with 50 mg/kg LG, 0 0.36 ml/kg BB; (**F**) MEs with 100 mg/kg LG, 0 0.36 ml/kg BB; (**G**) MEs with 200 mg/kg LG, 0 0.36 ml/kg BB; (**H**) and MEs with 400 mg/kg LG, 0 0.36 ml/kg BB.

Tissue from saline-treated mice shown in Fig. 2A exhibits healthy ultrastructure, as expected. Likewise, hepatic tissue from mice treated with 400 mg/kg LG alone (2B) and the MEs vehicle alone (2C) appear healthy, also as expected. By contrast, examination of slides made from livers of mice receiving BB revealed pathological changes in tissue architecture. These effects are most pronounced in livers from mice receiving BB but no LG (2D). A dose-responsive decrease in pathological changes in hepatic tissue architecture was observed in examination of mice receiving LG at 50 mg/kg (2E), 100 mg/kg (2 F), 200 mg/kg (2 G), and 400 mg/kg (2 H).

## 4 Discussion

The major outcomes of the present study include a clear demonstration that orally administered LG significantly suppresses BB-induced increases in serum activity of enzymes that correlate with liver toxicity: AST, ALT, ALP (Table 1), and hepatocellular injury, as demonstrated by histological analyses (Fig. 2). Although the data shown here do not allow an in-depth mechanistic interpretation of the results, toxicological studies of yield some insight into the observed effects of BB on mouse livers in the present investigation, and into the capacity of LG to counteract them. BB is a well-known hepatotoxin, widely used to induce experimental liver injury [20].

Metabolism of BB generates reactive species leading to oxidative stress and consequent hepatocellular damage [21], the extent of which may be easily monitored as a direct correlate of serum AST, ALT, and ALP activities [22].In the present study, elevated serum AST, ALT, and ALP activities observed after BB treatment (Table 1) are most likely due primarily to toxicant-mediated hepatocellular damage, demonstrated by histological examination (Fig. 2).The ability of LG to stimulate the observed protective effects reported here is an outcome that was expected, based on the known performance of the product [23,24], and on previous studies of the efficacy of milk thistle extract in treatment of liver disease [4].

The physiological mechanisms underlying the observed effect may be estimated by considering the nature of cytoprotective phytochemicals produced by the plant. The best known bioactive compound in milk thistle is silibinin, a polyphenol produced by the plant with strongly hepatoprotective and anti-tumor properties [25,26]. Milk thistle also contains structurally related polyphenols called (flavonolignans), including silydianin, silychristin, isosilychristin, the silybins and isosyilibins, and taxifolin, which is a highly potent cytoprotective flavonoid [27].

Microemulsions exhibit several advantages as a drug delivery system for instance, these are thermodynamically stable, require low energy level for formation, easy of manufacturing and improve drug solubility, bioavailability and efficacy. The aim of this work is to evaluate the hepatoprotective effect of LG loaded in MEs and to increase the oral bioavailability of LG in mice. Based on the results the efficacy of LG in MEs showed better drug solubility and permeability which lead to improve drug absorption among different biological membranes.

Due to very small particle size of MEs there would be high contact surface area between drug and gastrointestinal tract media. In previous studies [14,[28], [29], [30], [31]] we investigated MEs penetration through different biological membranes due to very small particle size of MEs and their biocompatibility with cellular barriers [32]. Poor effect of ME formulations on hepatic enzymes activity have been proved that these formulations are not toxic and oral bioavailability of LG could be increased via this carrier system.

According to our result, the hepatoprotective effect of this formulation against BB toxicity has been conducted through the control release, high diffusion and absorption rates and improve and increase in oral bioavailability of active pharmaceutical agents.

Histopathology examination of livers showed that BB induced severe damages in tissue architecture, including swelling of hepatic cytoplasm, steatosis centrilobular, necrosis, inflammation, and congestion. Treatment with LG has improved hepatic damages due to BB severe degeneration and vacuolation of hepatocytes. LG administration can probably repair the hepatic tissue through the stimulation of protein synthesis and hepatocytes regeneration.

The results of the present study show that LG is a potent cytoprotectant agent with potential for broad clinical use. The ability of this formulation to significantly suppress expression of enzymes elevated in response to toxigenic damage, and also inhibit damage to liver tissue ultrastructure in BB-treated mice, demonstrates its medical value. An intriguing corollary use for this product is in preventive medicine. There are negligible adverse side effects associated with human consumption of Livergol. For this reason, it might be given prophylactically to persons at risk for toxic liver damage of bromobenzene.

## Conflict of interest

The authors have no conflict of interest to disclose.

## Declaration of interests

This study was supported by grant no u-91134 from the Deputy of Research of Ahvaz Jundishapur University of Medical Sciences, Ahvaz, Iran. The authors are sincerely grateful to Ildikó Bácskay^2^ for her hard work in organizing, formatting, and editing this article.
